# Improvement of scoring system used before discharge to predict 30-day all-cause unplanned readmission in geriatric population: a prospective cohort study

**DOI:** 10.1186/s12877-024-04875-9

**Published:** 2024-03-25

**Authors:** Siti Setiati, Laurentius Johan Ardian, Ika Fitriana, Muhammad Khifzhon Azwar

**Affiliations:** 1 Division of Geriatrics, Department of Internal Medicine, Faculty of Medicine, Universitas Indonesia-Cipto Mangunkusumo Hospital, Jakarta, Indonesia; 2 Department of Internal Medicine, Faculty of Medicine, Universitas Indonesia-Cipto Mangunkusumo Hospital, Jakarta, Indonesia

**Keywords:** Readmission, Geriatric syndrome, Older adult, Malnutrition, Delirium

## Abstract

**Background:**

Data taken from tertiary referral hospitals in Indonesia suggested readmission rate in older population ranging between 18.1 and 36.3%. Thus, it is crucial to identify high risk patients who were readmitted. Our previous study found several important predictors, despite unsatisfactory discrimination value.

**Methods:**

We aimed to investigate whether comprehensive geriatric assessment (CGA) -based modification to the published seven-point scoring system may increase the discrimination value. We conducted a prospective cohort study in July–September 2022 and recruited patients aged 60 years and older admitted to the non-surgical ward and intensive coronary care unit. The ROC curve was made based on the four variables included in the prior study. We conducted bivariate and multivariate analyses, and derived a new scoring system with its discrimination value.

**Results:**

Of 235 subjects, the incidence of readmission was 32.3% (95% CI 26–38%). We established a new scoring system consisting of 4 components. The scoring system had maximum score of 21 and incorporated malignancy (6 points), delirium (4 points), length of stay ≥ 10 days (4 points), and being at risk of malnutrition or malnourished (7 points), with a good calibration test. The C-statistic value was 0.835 (95% CI 0.781–0.880). The optimal cut-off point was ≥ 8 with a sensitivity of 90.8% and a specificity of 54.7%.

**Conclusions:**

Malignancy, delirium, length of stay ≥ 10 days, and being at risk of malnutrition or malnourished are predictors for 30-day all-cause unplanned readmission. The sensitive scoring system is a strong model to identify whether an individual is at higher risk for readmission. The new CGA-based scoring system had higher discrimination value than that of the previous seven-point scoring system.

## Background

Older adults consistently had the highest rate of hospitalization among other age groups [1]. The 30-day readmission rate in ≥ 65 years population ranged from 11 to 23% in the United States [2]. Meanwhile, data from tertiary referral hospitals in Indonesia suggested a much higher rate ranging between 18.1 and 36.3% [3–5.

Rehospitalization may adversely affect patients, families and may become burden on health insurance payers. Therefore, rehospitalization needs to be given special attention as approximately one out of ten re-hospitalized cases was potentially preventable. In the United States, the Hospital Readmission Prevention Program (HRPP) conducted by Medicare showed preventing 10% cases of rehospitalization can save around 1 billion US dollars [6]. One of the preventive strategies is identifying the predicting factors for the incidence of rehospitalization, thus the planned intervention may be cost-effective and precise because implementing various expensive interventions may not be feasible in certain healthcare institution. Previous studies suggested that transitional interventions in high-risk population led to absolute risk reduction of 30-day readmissions by 11 to 28% points [7].

Geriatric syndromes are of paramount importance in the care for older adults since it may reflect the complexity of geriatric cases and contribute to hospitalization, higher healthcare costs, and readmission rate [8]. Our recent study suggested that a seven-point scoring system applied in older inpatients taking into account malignancy, nutritional status, depression, and functional status may predict 30-day unplanned readmission with unsatisfactory discrimination score (C-statistic value 0.694) [5]. Thus, we modified by adding several other potential parameters, such as delirium during hospitalisation, and examine discharged hemoglobin and sodium level. Both of the laboratory result has been included as predictors in HOSPITAL readmission score study [9]. These laboratory parameters might reflect the early signal of changes, even though there were any changes clinically yet [10].

Anemia has become one of the common conditions bringing patients to be rehospitalized, especially in cancer patients [11, 12]. Lau et al. [13] also found chronic anemia due to haemorrhage could predict rehospitalisation in older patients (OR = 2,4 *p* < 0,0001). Besides anemia, hyponatremia also often became highly prevalent in hospitalized older adults, Lu et al. [14] found untreated and persistent discharged hyponatremia increased significant risk of readmission in hospital (OR: 1.41 (95% CI 1.17–1.71), *p* < 0.001).

We aimed to conduct a prospective cohort study to investigate whether of those modifications to the published seven-point scoring system may increase the discrimination value in order to create a better discrimination value involving different study population.

## Methods

### Study design and subjects

We recruited older adults aged ≥ 60 years admitted to non-surgical wards and intensive coronary care unit (ICCU) in CiptoMangunkusumo Hospital in July-September 2022. This prospective cohort study utilized comprehensive geriatric assessment (CGA) done at the end of the hospital admission among older adults who agreed to participate in the study. Patients with severe loss of consciousness (Glasgow Coma Scale < 8) were not recruited. We did 30-day follow-up after discharge using a phone call to collect readmission data. We excluded patients who could not be reached during follow-up session. We also excluded patients who died during follow-up period and the family who decided not to bring them to the hospital.

### Baseline data and follow up

We collected the baseline data using questionnaires within three days before hospital discharge. The data related to delirium during admission, length of stay, polypharmacy, serum sodium and haemoglobin level were collected from medical records. Readmission in this study was defined as admission to an acute care hospital within 30 days of discharge from an acute care hospital. We determined the outcome as all-cause unplanned readmissions [15].

The data from history taking and medical record included (i) demographic data (sex, age, educational background); (ii) functional status based on the Barthel ADL index questionnaire, categorized into two categories, namely functional independence & mild functional dependence (score ≥ 12), and moderate– total functional dependence (score < 12); (iii) nutritional status based on Mini Nutritional Assessment short-form (MNA-SF) to classify patients into two groups, namely being not malnourished (MNA-SF ≥ 12), and being at risk of malnutrition or malnourished (MNA-SF < 12); (iv) cognitive function based on the Abbreviated Mental Test (AMT) questionnaire with two result classifications, namely cognitive impairment (score 0–6) and normal (score 7–10); (v) depression was based on the 15-item Geriatric Depression Scale (GDS-15), result of which helped classify patients into normal patients (GDS-15 < 4) and patients at risk of depression (GDS-15 ≥ 4); (vi) Charlson Comorbidity Index (CCI), cross-sectionally taken from interview questionnaire and medical records for the last one year, the results of which were categorized into mild and moderate (score 0–4), as well as severe (score ≥ 5); (viii) polypharmacy, which was defined as the administration of five or more medications daily [16]; (ix) history of previous admission in the previous 6 months; (x) haemoglobin level at discharge (mg/dL); (xi) serum sodium level at discharge (mEq/L); (xii) delirium during admission; (xii) the length of hospital stay, which was defined as the patient’s duration of whole hospital stay from emergency admission until hospital discharge, neither included outpatient visits nor limited to acute ward stay period; and (xiii) the presence of malignancy.

The diagnosis of delirium in our study was made by licensed physicians based on the confusion assessment method (CAM) [17]. It is the most commonly advocated method in clinical pathways and guidelines globally, as well as most commonly used in clinical studies [18]. The method was chosen due to its moderate-to-high specificities ranging between 84% and 100%. We categorized delirium types in descriptive data into hyperactive, hypoactive, and mixed type [19]. Hyperactive delirium was characterized by restlessness, motor agitation, and sometimes aggressiveness, whereas hypoactive delirium was characterized by slowed speech, apathy, motor retardation, and individuals may appear to be sedated. Mixed type was defined as the combination of hypoactive and hyperactive delirium.

### Statistical analyses

We calculated the sample size using the rule of thumb and the equation for comparing two areas under the curve (AUCs) [20]. We utilised two-way hypothesis test. The value of alpha and beta were 5% and 10%, respectively.

We utilised SPSS software version 24.0 (IBM, Armonk, New York, United States of America) to analyse our research data. First, we provided descriptive report of our data. Variables included in the prior seven-point system score, consisting of malignancy, malnutrition, dependent functional status, and depression, were then given a score based on the previous study conducted by Fitriana et al. [21]. Then we determined the C-statistic value based on that category. For analytical purposes, the independent variables were categorized into dichotomous variables. Cut-off points from the prior study were used for malnutrition and depression variables. Moreover, the cut-off points of other numeric variables, such as length of stay, dependency, sodium, and hemoglobin level, was determined using the Receiver Operating Characteristics (ROC) curve. These variables were then categorized based on the intersection points obtained [20]. We used Pearson’s correlation and VIF values for collinearity and linear correlation tests. We used chi-square for bivariate analysis to obtain the association between independent variables and 30-day unplanned readmission. It was followed by multivariate analysis using stepwise multiple logistic regression for all variables with p-values < 0.25 in the bivariate analysis result to obtain the prognostic factors for 30-day all-cause unplanned readmission.

Scores were obtained from Beta/Schneeweiss method. Weights were increased by 1 unit with each 0.3 increase in the β [21]. We calculated the total risk score and analysed the C-statistic value. The optimal cut-off point value were found using the ROC curve. We also obtained the equation from the total score results to find the probability value of the outcome. We used Hosmer-Lemeshow calibration test to test the goodness of fit of the logistic regression model. Furthermore, We utilised bootstrapping method with *n* = 1000 for internal research validation.

We compared the results of the AUC values ​​with the AUC results obtained when only four variables were included in the original seven-point scoring system as published in prior study. We utilized De Long method in MedCalc version 20.115 (MedCalc Software Ltd, Flanders, Belgium) for the comparative study of AUC values.

### Ethical approval

Ethical approval was obtained from the Research Ethics Committee at the Faculty of Medicine, Universitas Indonesia. (No. KET-639/UN2.F1/ETIK/PPM.00.02/2022).

## Results

Initially, 242 patients recruited during this period met the inclusion criteria of our study. During follow-up period, we excluded 7 patients, consisting of 3 patients who could not be contacted during follow-up and 4 patients who died at home, and the family decided not to take the patient to the hospital.

Of the 235 subjects who completed the study, 136 (57.87%) were male, and the median (range) age was 66 (60–94) years. Thirty-eight subjects (16.1%) had delirium, and 87 (37.02%) had malignancy. Lengths of stay ranged from 2 to 30 days with a median of 10 days, see Table 1. We have declared no missing data in this study. Readmission rate within 30 days was 32.3% (95% CI 26–38). The calibration test using the rule in Fitriana et al. [5] of the four known predictors was carried out. We found that the AUC value was 0.734 (0.672–0.789).

**Table 1:** Baseline characteristics of subjects

Characteristics	Subjects (*n* = 235)
Age, median (min-max), years	66 (60–94))
Sex (n, %)	
Male	136 (57.87)
Female	99 (42.12)
Formal education completion (n, %)	
No formal education completion	14 (5.96)
Primary school	47 [20]
Junior high school	34 (14.47)
Senior high school	89 (37.87)
Higher education	48 (20.42)
Length of stay, median (Min-Max), days	10 [2–30]
History of delirium during admission	
Yes	38 (16.1)
No	197 (83,8)
Delirium type (*n* = 38)	
Hyperactive	14 (36.8)
Hypoactive	12 (31.6)
Mixed type	12 (31.6)
Charlson Comorbidity Index (CCI)	
CCI < 5	113 (48)
CCI ≥ 5	122 (52)
History of falls during previous 6 months (n, %)	
Yes	39 (16.56)
No	196 (83.4)
Malignancy	
Yes	87 (37.02)
No	148 (62.98)
Metastasis (*n* = 87)	
Yes	35 (40.2)
No	46 (52.9)
Hematological malignancy	6 (6.9)
Malignancy type (*n* = 87)	
Hepatic malignancy	15 (17.2)
Lung malignancy	7 [8]
Cervical malignancy	7 [8]
Cholangiocarcinoma	4 (4.6)
Gastrointestinal malignancy	6 (6.9)
Nasopharyngeal malignancy	4 (4.6)
Leukaemia	3 (3.4)
Lymphoma	2 (2.3)
Others	25 (40.2)
History of prior admission (n, %)	
Yes	130 (55.32)
No	105 (44.68)
Depression (n, %)	
Normal	174 (74.04)
At risk of depression	61 (25.96)
Cognitive function (n, %)	
Cognitive impairment	30 (12.8)
No cognitive impairment	205 (87.2)
Nutritional status (n, %)	
Not malnourished	52 [22]
At risk of malnutrition or malnourished	183 (77.9)
Functional status (n, %)	
Functional independence & mild functional dependence	172 (73.20)
Moderate– total functional dependence	63 (26.80)
Haemoglobin at discharge (mg/dL) median (min-max)	10.8 (7.6–17.1)
Serum sodium at discharge (mEq/L) median (min-max)	135 (121–143)

We determined the cut-off point of several numeric variables from intersection point of the ROC curve. The cut-off point of 10 was chosen for the length of stay variable, 10 for the hemoglobin variable, 135 for the sodium variable, and 16 for the dependency variable. Bivariate analysis results in Table 2 showed that several covariates, namely nutritional status, malignancy, functional status, length of stay, and delirium, had a significant relationship with our outcome, so that it could be included in multivariate analysis. Multivariate analysis results suggested statistically nonsignificant result for functional status, see Table 3. The adjusted Odds Ratio (OR) (95% CI) for malignancy was 5.19 (2.955–11.833), whereas it was 3.252 (1.365–7.746) for delirium, 8.919 (1.992–39.930) for being at risk for malnutrition– malnourished, 0.789 (0.354–1.762) for moderate– total functional dependence, and 3.081 (1.501–6.324) for length of stay ≥ 10 days.

**Table 2:** Bivariate analysis result

Independent Predictor	Readmission		P value
	Yes	No	
	(*n*=76)	(*n*=159)	
Malignancy (n, %)			
Yes	50 (57.5)	37 (42.5)	<0.001
No	26 (17.6)	122 (84.2)	
Nutritional status			
Normal	74 (40.4)	109 (59.6)	<0.001
At risk malnoursihed– malnourished	2 (3.8)	50 (96.2)	
Functional status (n, %)			
Functional independence & mild functional dependence	45 (26.2)	127 (73.8)	0.001
Moderate– total functional dependence	31 (49.2)	32 (50.8)	
Depression			
Normal	57 (32.8)	117 (67.2)	0.368
At risk of depression	19 (31.1)	42 (68.9)	
Length of stay (n, %)			
< 10 days	16 (14.5)	94 (85.5)	<0.001
≥ 10 days	60 (48)	65 (52)	
Delirium history (n, %)			
Yes	22 (57.9)	16 (42.1)	<0.001
No	54 (27.4)	143 (72.6)	
Haemoglobin at discharge (mg/dL) (n, %)			
< 10 mg/dL	18 (27.7)	47 (72.3)	0.346
≥ 10 mg/dL	58 (34.1)	112 (65.9)	
Serum sodium at discharge (mEq/dL) (n, %)			
< 135 mEq/dL	33 (32.4)	69 (67.6)	0.997
≥ 135 mEq/dL	43 (32.3)	90 (67.7)	

**Table 3:** Multivariate analysis result

Variable	Adjusted OR (95% CI)	P value
Malignancy		
Yes	5.19 (2.955– 11.833)	<0.001
No (reference)	1	
Delirium		
Yes	3.252 (1.365– 7.746)	0.008
No (reference)	1	
Nutritional status		
Not malnourished (reference)	1	0.004
At risk for malnutrition– malnourished	8.919 (1.992– 39.930)	
Functional status (n, %)		
Functional independence & mild functional dependence (reference)	1	0.564
Moderate– total functional dependence	0.789 (0.354– 1.762)	
Length of stay		
<10 days (reference)	1	0.002
≥10 days	3.081 (1.501– 6.324)	

**Fig. 1:**
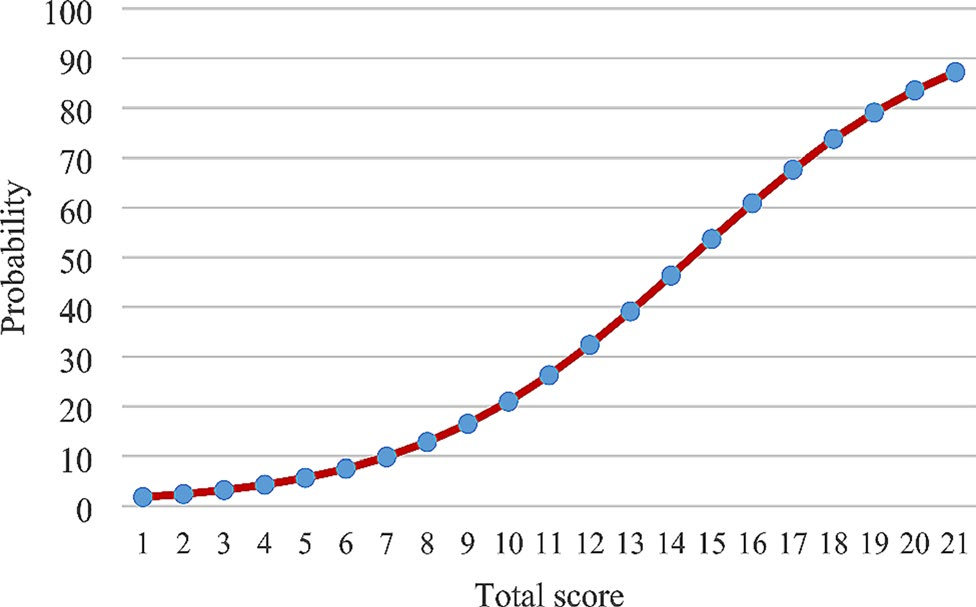
Probability of outcome based on total score equation

**Fig. 2:**
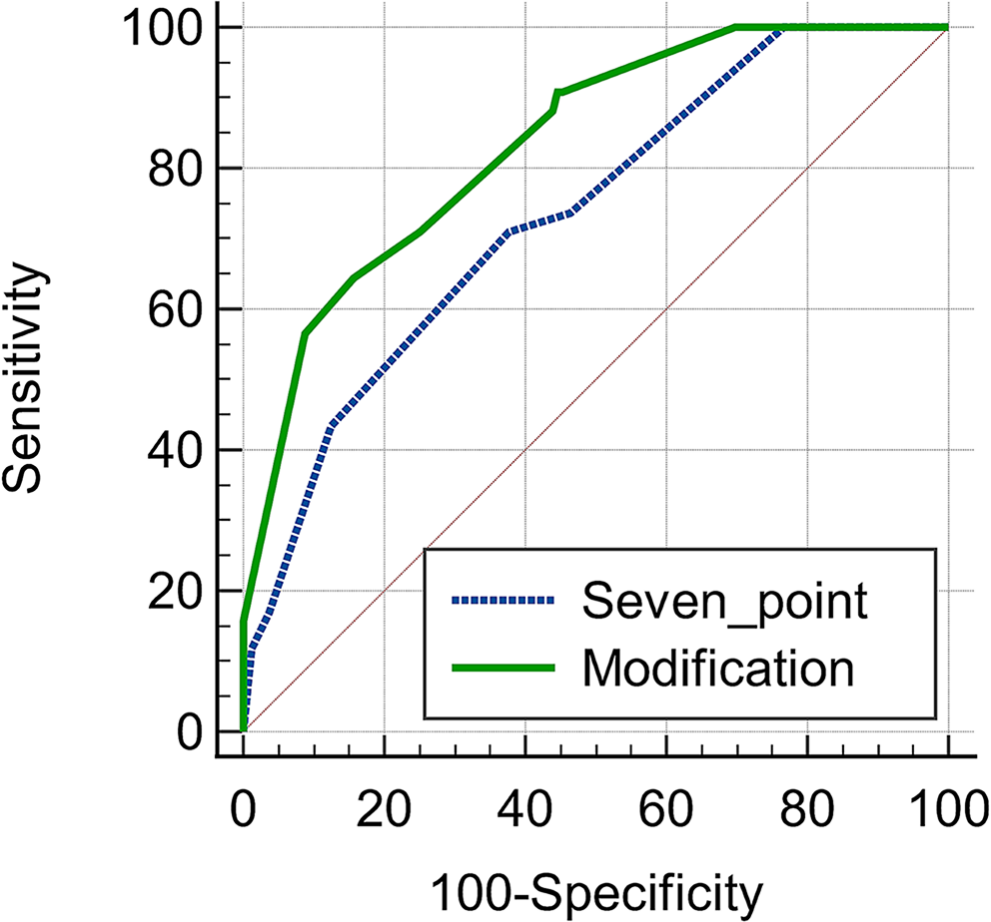
Comparison between Receiver Operating Characteristics (ROC) of the original seven-point scoring system and the modified scoring system to predict 30-day all-cause unplanned readmission in older adults

In the final model, several variables were identified in the development of a new scoring system, see Table 4. The scoring system had maximum score of 21 and incorporated malignancy (6 points), delirium (4 points), length of stay ≥ 10 days (4 points), and being at risk of malnutrition or malnourished (7 points). The greater the score an individual has, the higher chance the individual is readmitted, see Fig. 1. From this scoring system, a cut-off point of 8 had 90.8% sensitivity and 54.7% specificity. (Table 5) The C–statistic value was 0.835 (95% CI 0.781–0.880). The calibration test of the prediction model obtained was carried out using the Hosmer-Lemeshow test with a significance value of *p* = 0.249 (*p* > 0.05). (Fig. 2).

**Table 4:** Derivation of scoring system to predict 30-day unplanned all-cause readmission from stepwise multivariate analysis (*n* = 235)

Variable	B coefficient	SE	P value	Score
Malignancy	1.777	0.354	< 0.001	6
Delirium	1.179	0.443	0.007	4
Length of stay ≥ 10 days	1.125	0.367	0.002	4
At risk of malnutrition or malnourished	2.188	0.765	0.004	7
Constant	-4.389	0.791	< 0.001	

**Table 5:** Prognostic test characteristic of scoring model for readmission

	Sensitivity	Spesificity	PPV	NPV
≥ 4	100%	18.23%	36.9%	100%
≥ 8	90.8%	54.7%	48.9%	92.6%
≥ 11	88.2%	56%	48.9%	90.8%
≥ 13	71.1%	74.8%	57.4%	84.4%

## Discussion

In this prospective cohort study, we enhanced the seven-point scoring system publishedbeforehand [5]. The components of the newly enhanced scoring system were malignancy, delirium, length of stay ≥ 10 days, and being at risk of malnutrition or malnourished (MNA-SF < 12).

Our new scoring system had a higher AUC score of 0.835 (95% CI 0.781–0.880) than the four variables in the previously published seven-point scoring system. AUC score of 0.835 may signify very good test quality of this scoring system. This modified score has a moderate discrimination test with good calibration, with C–statistic value being 0.835. Thus, the newly modified scoring system has a good calibration performance to predict the readmission of geriatric patients. The two AUC curves of the two scoring systems were significantly different (*p* < 0.0001). The greater the score an individual has, the higher probability that the individual will be readmitted.

The optimal cut-off point was ≥ 8 with a sensitivity of 90.8% and a specificity of 54.7%. (Table 5) This implied 90.8% readmitted patients had a score ≥ 8 or had at least 2 predictors. Meanwhile, 54.7% of patients who were not readmitted has a score < 8 or only 1 predictor. Such high sensitivity indicates that we could confidently rule out possible 30-day all-cause unplanned readmission in geriatric population with total score of < 8. On the other hand, suboptimal specificity may mean that our scoring system potentially result in high rate of false negative cases [22]. From the PPV and NPV result showed older adults discharged \with a score of 8 or higher have 48.9% the possibility of undergoing treatment again. Meanwhile, if the score is < 8, they have 92.6% chance of not undergoing readmission.

Because the C-statistic value of our improved scoring system exceeded 0.8 [23], the model was considered strong to identify whether or not an individual is at higher risk for 30-day all-cause unplanned readmission. Compared to the existing scoring system, the C-statistic value of our current study is better than LACE [24], HOSPITAL [9], PARA [25], RRS [26] score, and the score published by Tsui and colleagues [27]. However, our c-statistic value was less lower than that of SEMI score [28]. Because there were multiple factors linked to readmission in geriatric population, focusing on the geriatric syndrome(s) and co-morbidities rather than individual diseases only in geriatric patients might generate a promising result [8]. The recent systematic review by Zhou et al. [29] including 60 studies with 73 unique predictive models met the inclusion criteria suggested that only a few studies included geriatric giants, in their research. Moreover, the geriatric giants such as nutritional problem was only assessed on the basis of body mass index (BMI), which might not be an appropriate tool for older adults.

Our readmission rate in this study is higher than in other studies mentioned. This might be related to the unique characteristics of subjects in our study and various definitions of readmission among scientific studies in the past. All-cause readmission was chosen in this study as the outcome rather than readmission caused by index admission due to several reasons. Firstly, from the patient’s perspective, readmission for any reason is not the expected healthcare outcome. Secondly, readmission for any reason exposes the patient to risks associated with hospitalization, such as iatrogenic causes. Secondly, there is no reliable way to determine whether readmission is related to causes documented in prior treatment. Thirdly, the all-cause readmission criteria are consistent with the indicators used by Medicare in reporting readmission events [15].

Malnutrition and malignancy consistently became significant predictors of readmission. The malnutrition assessment tool that we used, MNA-SF, strongly correlated with MNA score, and was 97.9% sensitive and 100% specific to predict undernutrition, with overall diagnostic accuracy of 98.7% [30]. Nutrition is crucial for post-hospital patients when the body’s physiological systems are still disturbed and have not fully recovered. Malnutrition conditions may adversely affect wound healing, increase the risk of infection and pressure sore, decrease respiratory and heart function, cause cardiovascular and gastrointestinal disorders, and worsen functional status of an individual [31]. Patients with malignancy have the tendency to have readmission due to the tendency to suffer infections. Tennison et al. [32] suggested that the most frequent causes of patients with cancer undergoing rehospitalization included infections at 7.8% and malignancy-related conditions at 3.5% with a median treatment time of 14 days.

Length of stay was a significant predictor of readmission in our study. The longer length of stay reflects the complexity of the disease and may expose patients to a higher risk of nosocomial infection and deconditioning [25]. Delirium was also a significant predicting factor. The incidence of delirium can reduce a person’s cognitive function and functional status. Following hospital discharge, individuals with delirium may become challenging to treat at home. It may also trigger aspiration, reduction in oral intake, and reduction in mobility. It could also increase the incidence of pulmonary embolism [33].

The data analysis in our study suggested statistically nonsignificant results related to two laboratory test results involved in our study, namely low haemoglobin and low serum sodium. Anemia may adversely impact the readmission rate in previous studies. However, this was only found in a few groups of patients, such as patients with heart failure [34]. On the other hand, there was no relationship between haemoglobin levels at discharge and the incidence of rehospitalization among geriatric patients who had trauma [35] and chronic kidney disease [36]. Furthermore, Potasso et al. [37] found that sodium levels at discharge had an effect on the incidence of recurrence of pneumonia, but had no effect on the outcome of rehospitalization and mortality.

Our study had several strengths. First, the prospective cohort method may provide evidence suggesting causal relationship and information about the strength of the relationship between risk factors and outcome. Second, the multivariate logistic regression analysis may stratify the relationship between and minimize bias. Third, this study was conducted at the end of treatment, which best describes the patient’s profile before discharge. Fourth, this study presented a newly improved scoring system based on CGA that is practical to use in daily clinical practice. This newly improved scoring system may be used in clinical practice. Through assistance, various interventions, including post-discharge interventions such as home visits, communication channels, counseling, and nutritional supplementation, can be attempted, all of which are carried out to improve the patient’s clinical condition so that rehospitalization incidents can be prevented [38].

We acknowledge the limitations of the present study. First, this research was carried out in a single tertiary referral hospital. Therefore, it is necessary to conduct studies to evaluate this scoring system in lower-level hospitals, if possible in the form of multicenter study with larger sample size. Second, unlike 4 As Test (4AT), CAM that we used in our study may have variable sensitivities (9-100%) as suggested by the assessment of 23 studies in the past [19, 39, 40]. CAM was chosen in this study due its simplicity and high sensitivities. Moreover, 4AT is not the most commonly used in clinical practice in the region. For this reason, it is necessary to consider other factors that are difficult to measure but contribute to individuals undergoing hospitalization again, such as readiness of patient and family of being discharged and quality of transition care.

## Conclusions

In conclusion, the improved scoring system incorporated malignancy, delirium, length of stay ≥ 10 days, and being at risk of malnutrition or malnourished (MNA-SF < 12). The new CGA-based scoring system had higher discrimination value than that of the previous seven-point scoring system to predict 30-day all-cause unplanned readmission. This modified score has a moderate discrimination test with good calibration. The optimal cut-off point was ≥ 8 with a sensitivity of 90.8% and a specificity of 54.7%.

### Ethical approval

## Data Availability

The datasets used and/or analysed during the current study are available from the corresponding author on reasonable request.

## Abbreviations

4AT: 4 As Test
AMT: Abbreviated Mental Test
AUC: Area under the curve
CAM: confusion assessment method
CCI: Charlson Comorbidity Index
CI: confidence interval
CGA: comprehensive geriatric assessment
GDS-15: 15-item Geriatric Depression Scale
ICCU: intensive coronary care unit
MNA-SF: Mini Nutritional Assessment short-form
ROC: Receiver Operating Characteristics
